# Blocking Air in the Filtering Bleb: Effects on Graft Adhesion After Descemet’s Stripping Automated Endothelial Keratoplasty

**DOI:** 10.7759/cureus.67459

**Published:** 2024-08-22

**Authors:** Keisuke Iwakawa, Koichiro Shinji, Naho Kurisu, Sosuke Inokawa, Tai-ichiro Chikama

**Affiliations:** 1 Ophthalmology, Hiroshima University, Hiroshima, JPN; 2 Ophthalmology, Saiseikai Kure Hospital, Kure, JPN; 3 Ophthalmology, Hiroshima Prefectural Hospital, Hiroshima, JPN; 4 Ophthalmology, Miyoshi Central Hospital, Miyoshi, JPN; 5 Ophthalmology, Inokawa Eye Clinic, Kure, JPN

**Keywords:** endothelial graft adhesion, intraocular pressure, filtering bleb, descemet’s stripping automated endothelial keratoplasty, blocking air

## Abstract

Background: To evaluate the outcome of Descemet’s stripping automated endothelial keratoplasty (DSAEK) with “blocking air” in the filtering bleb in patients with previous trabeculectomy.

Methods: In total, 299 eyes in 283 patients who underwent DSAEK were retrospectively reviewed. Endothelial graft adhesion, intraocular pressure (IOP), and air volume in the anterior chamber with (group A) or without (group B) a filtering bleb were compared between the groups. Group A was divided into two subgroups according to the presence (group A1) or absence (group A2) of air in the filtering bleb; the same three factors were compared between the subgroups.

Results: The graft detachment rate was significantly higher in group A (14.3%) than in group B (6.5%) (p = 0.04). IOP was significantly lower in group A than in group B before surgery (p = 0.01), at the end of surgery (p = 0.04), at three hours (p < 0.001), and one week postoperatively (p = 0.02). The graft detachment rate did not significantly differ between groups A1 and A2. There were no differences in IOP at each follow-up time, whereas there was a statistically significant increase in IOP from the preoperative measurement to the end of surgery in group A1 (21.0±7.0 mmHg) compared with group A2 (14.2±8.6 mmHg) (p = 0.02).

Conclusions: The presence of blocking air in the filtering bleb helps ensure increased IOP during the early postoperative period but had no significant effect on graft detachment rates.

## Introduction

Descemet’s stripping automated endothelial keratoplasty (DSAEK) is widely applied to treat bullous keratopathy. Early postoperative complications of DSAEK include graft detachment, endothelial graft rejection, primary graft failure, iatrogenic glaucoma, and corneal endothelial cell loss, among which graft detachment is the most common complication [1-4]. Excessive surgical manipulation and poor-quality donor grafts adversely affect graft adhesion by inhibiting endothelial pump function [5]. In addition, low intraocular pressure (IOP) allows fluid to enter the graft interface, leading to graft detachment [6].

High IOP is temporarily required to achieve graft adhesion after DSAEK. The presence of a filtering bleb tends to reduce IOP, resulting in graft detachment. A history of filtering surgery is associated with a risk for postoperative graft detachment after DSAEK [7-12]. Aqueous humor and air in the anterior chamber (AC) are presumed to readily escape into the filtering bleb after DSAEK.

When the AC is filled with air during surgery, some air migrates from the AC into the filtering bleb. This air remains in the filtering bleb for several days postoperatively. We hypothesized that air in the filtering bleb blocks the aqueous humor outflow and results in an increase in postoperative IOP. If this hypothesis is correct, the presence of air in the filtering bleb may favor graft adhesion. We investigated the frequency of graft detachment in eyes with a filtering bleb and the effect of the presence or absence of blocking air on IOP and the graft detachment rate.

## Materials and methods

This retrospective review included 299 eyes from 283 patients who underwent DSAEK at Hiroshima University Hospital from December 2013 through June 2019. The study protocol adhered to the tenets of the Declaration of Helsinki and was approved by the Institutional Review Board of Hiroshima University (E-709). Informed consent was obtained in the form of opt-out, where participants were provided with the study information and were informed that their participation would be assumed unless they chose to decline.

**Figure 1:**
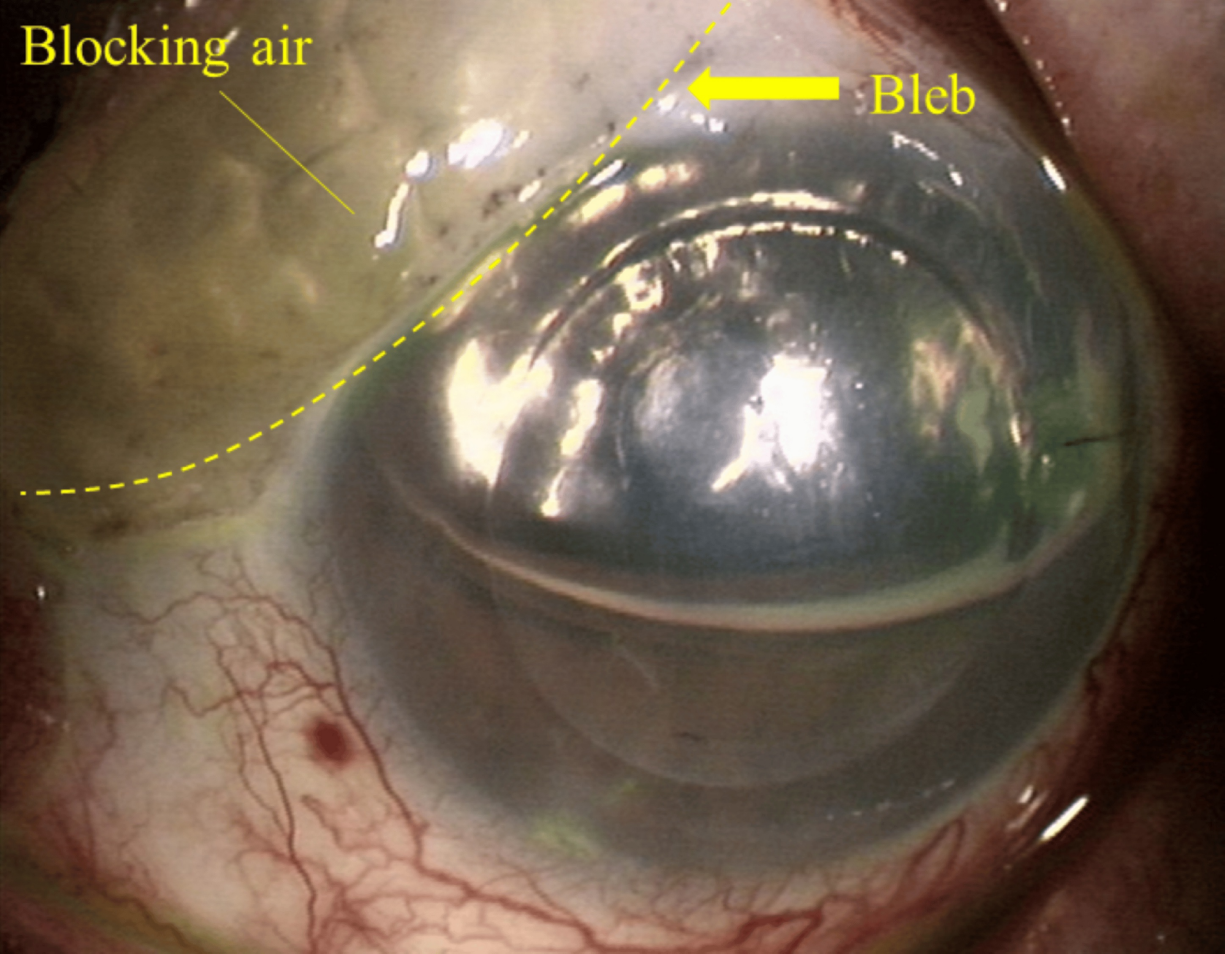
Slit-lamp image of blocking air in the filtering bleb. Air migrates into the filtering bleb when the anterior chamber is filled with air during Descemet’s stripping endothelial keratoplasty in the eyes with a filtering bleb.

We assigned the patients to two groups according to the presence (group A) or absence (group B) of a filtering bleb. It was confirmed by the medical record that the eyes of group A had a functional bleb. The graft detachment rate was recorded at one week postoperatively, and the IOP and air volume were determined in the AC, both preoperatively and in the early postoperative period (at three, six, and 24 hours, as well as one week and one month). The mean IOP at each time point and changes over time between the two groups were compared. Furthermore, group A was divided into two subgroups according to the presence (group A1) or absence (group A2) of air leakage into the filtering bleb. We have called air in the filtering bleb "blocking air" (Figure 1). The same three factors as above were evaluated for these subgroups. Subsequently, all patients were followed for a minimum of 1 month postoperatively.

Graft detachment was defined as a space present between the graft and the recipient’s corneal stroma according to slit-lamp examination findings. IOP was measured using the iCare tonometer (Tiolat Oy, Helsinki, Finland). The amount of air in the AC was defined as the proportion of air in the AC according to slit-lamp examination findings.

All statistical analyses were performed using JMP software, version 9 (SAS Inc., Cary, NC). Preoperative and postoperative parameters were compared between groups A and B and between groups A1 and A2. For all comparisons, the same outcome measures and continuous variables were compared using the Mann-Whitney U test for variables with skewed distributions. Finally, categorical variables were compared using the chi-squared test. A p-value of < 0.05 was considered to be statistically significant.

## Results

**Figure 2:**
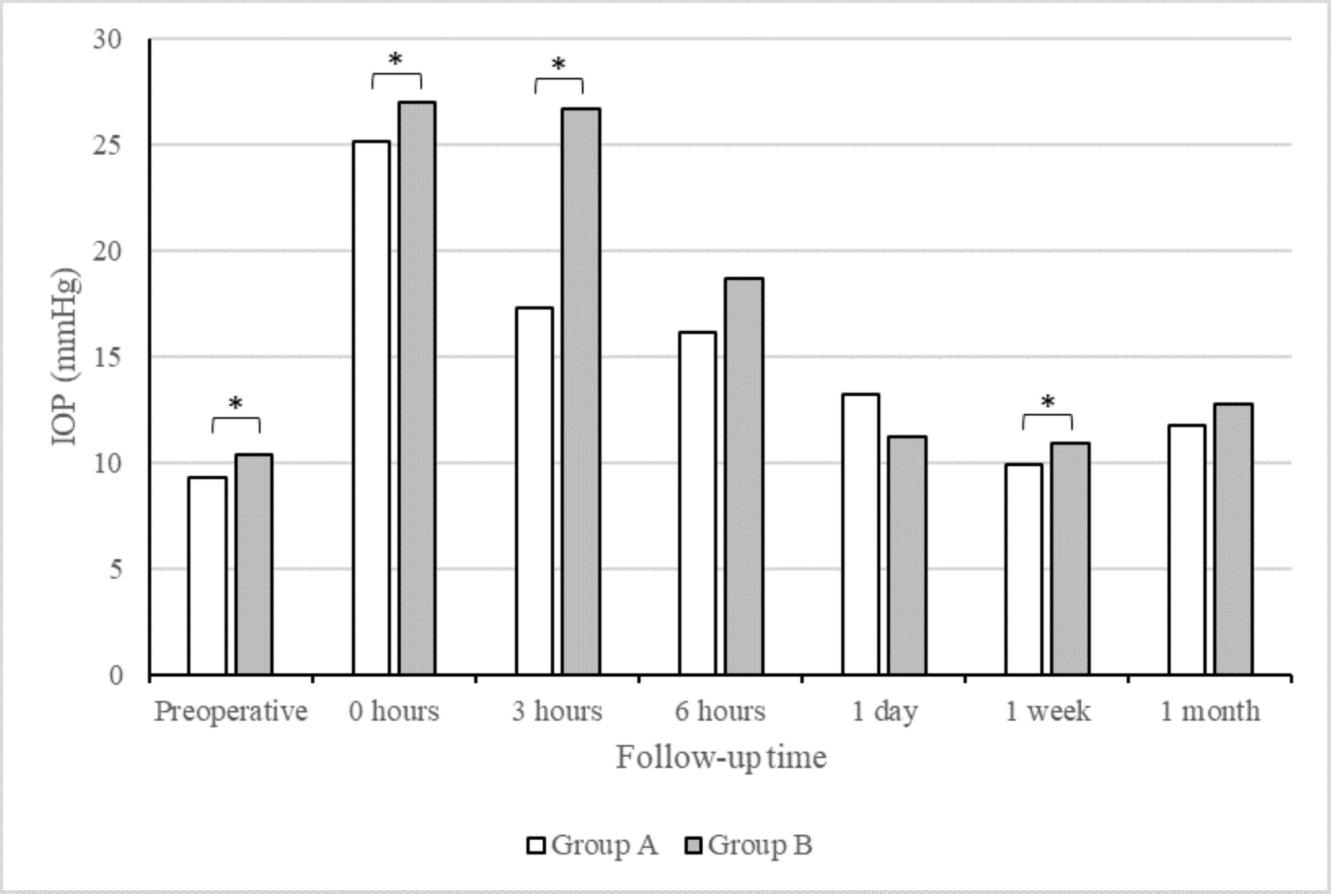
Intraocular pressure (IOP) recorded over time. IOP was significantly lower in group A than in group B before surgery, at the end of surgery, at three hours postoperatively, and at one week postoperatively (p = 0.01, 0.04, < 0.001, and 0.02, respectively). *p < 0.05

**Figure 3:**
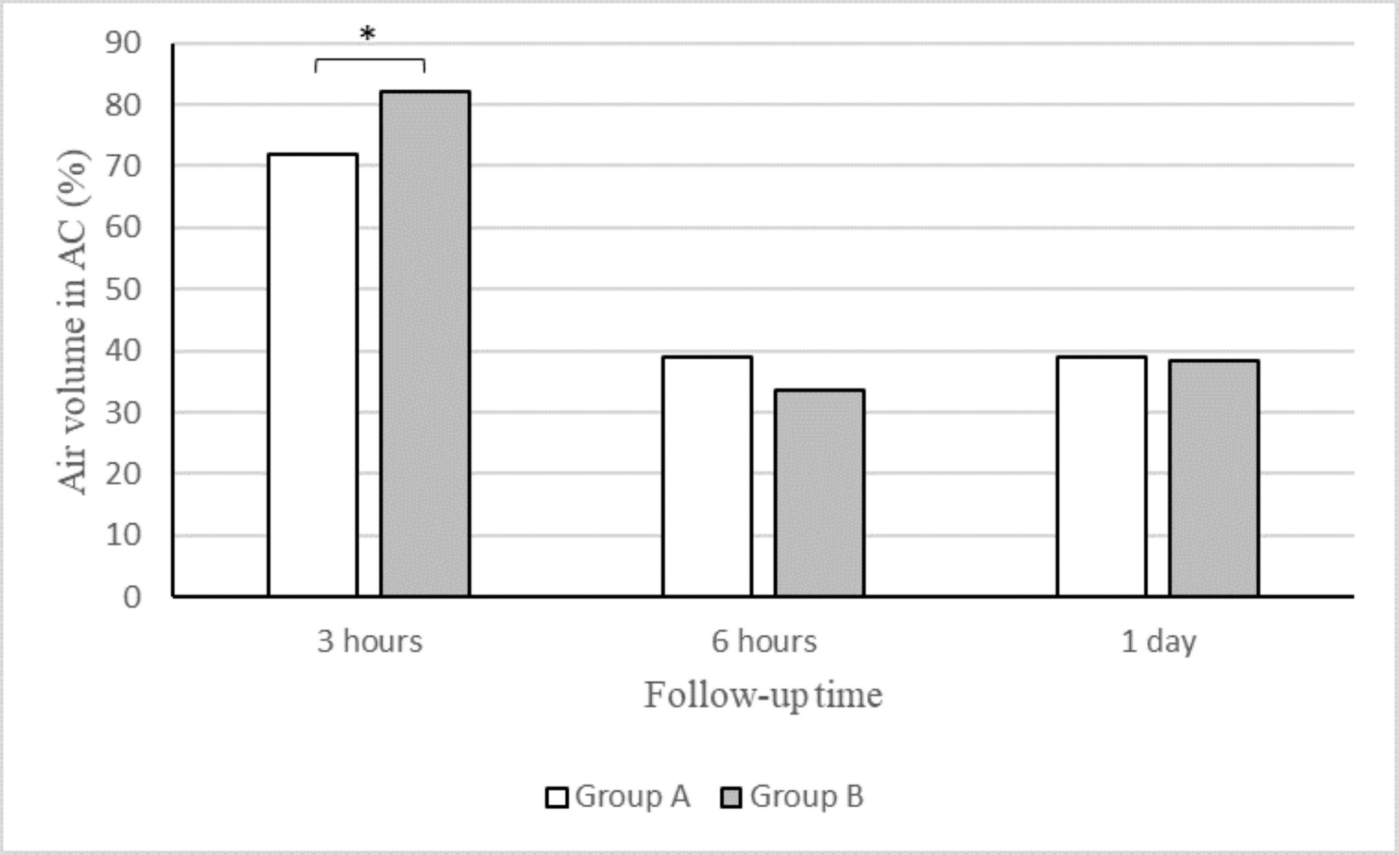
Air volume in the anterior chamber (AC) recorded over time. Air volume in the AC was significantly lower in group A than in group B at the end of surgery. *p < 0.05

The preoperative characteristics of the patients in groups A and B are presented in Table 1. Group A consisted of 85 eyes from 81 patients with previous trabeculectomy (TLE) who underwent DSAEK during the study period; group B comprised 214 eyes from 202 patients without previous TLE who underwent DSAEK surgery during the same period. There were no significant differences between groups A and B in age, sex, or lens status. The preoperative IOP was significantly lower in group A (9.3±4.9 mmHg) than in group B (10.4±4.0 mmHg) (p = 0.01). The preoperative characteristics of the patients in groups A1 and A2 are also shown in Table 1. Group A1 consisted of 16 eyes from 16 patients, while group A2 consisted of 69 eyes from 65 patients. There were no significant differences between groups A1 and A2 in age, sex, or lens status. The preoperative IOP was significantly lower in group A1 (7.0±3.5 mmHg) than in group A2 (9.8±5.0 mmHg) (p = 0.04). Table 2 shows the graft detachment rates in groups A and B, which were significantly higher in group A (14.3%, 12 eyes) than in group B (6.5%, 14 eyes) (p = 0.04). As shown in Figure 2, the IOP was significantly lower in group A than in group B at the end of surgery (25.2±7.5 vs. 27.0±7.5 mmHg) (p = 0.04) and at three hours (17.3±10.5 vs. 26.6±13.7 mmHg) (p < 0.001) and one week postoperatively (9.9±6.7 vs. 10.9±4.6 mmHg) (p = 0.02). As shown in Figure 3, the air volume in the AC was significantly lower in group A (71.9±30.3%) than in group B (82.3±24.9%) at three hours postoperatively (p = 0.001). By contrast, there were no significant differences in the air volume in the AC between the two groups at six hours or one day postoperatively.

**Table 1:** Preoperative characteristics in each group. Data are shown as the number (percentage) or mean ± standard deviation, unless otherwise indicated.

	Group A	Group B	P-value	Group A1	Group A2	P-value
No. of eyes	85	214		16	69	
Mean age (range), years	75.0±10.6 (44–93)	74.1±10.9 (38–95)	0.5	75.8±6.2 (64–85)	75.1±11.3 (44–93)	0.6
Sex (female)	49 (58%)	108 (53%)	0.3	8 (50%)	39 (57%)	0.6
Intraocular pressure (range), mmHg	9.3±4.9 (3–26)	10.4±4.0 (4–28)	0.01	7.0±3.5 (3–14)	9.8±5.0 (4–26)	0.04
Pseudophakia	81 (95%)	207 (97%)	0.6	15 (94%)	66 (96%)	0.7

**Table 2:** Comparison of graft detachment rates between groups A and B. Data are shown as the number, unless otherwise indicated.

	Group A	Group B	P-value
	(n = 85)	(n = 214)	
Adhesion of corneal grafts			
Attached	73	200	
Detached	12	14	
Graft detachment rate (%)	14.3	6.5	0.04

The graft detachment rate, IOP, and air volume in the AC were compared between groups A1 and A2. The graft detachment rate did not significantly differ between group A1 (25.0%, four eyes) and group A2 (11.6%, eight eyes) (p = 0.2). Furthermore, there were no significant differences between these two groups in the air volume in the AC after surgery (at three and six hours and one day; p = 0.3, 0.3, and 0.07, respectively). Table 3 shows the preoperative and postoperative IOP at each time point, together with changes between time points, for both groups. The change in IOP from the preoperative measurement to the end of surgery was significantly greater in group A1 (21.0±7.0 mmHg) than in group A2 (14.2±8.6 mmHg) (p = 0.02).

**Table 3:** Comparison of IOP between groups A1 and A2. IOP: intraocular pressure Data are shown as the mean ± standard deviation, unless otherwise indicated.

	Group A1	Group A2	P-value
	(n = 16)	(n = 69)	
Preoperative IOP (mmHg)	7.0±3.5	9.8±5.0	0.04
Postoperative IOP (mmHg)			
0 hours	27.9±9.4	24.5±6.8	0.2
3 hours	19.1±12.1	16.9±10.0	0.6
6 hours	18.3±9.6	15.6±7.5	0.3
1 day	12.1±4.5	13.4±7.2	0.7
1 week	7.9±4.9	10.4±7.1	0.2
1 month	8.6±4.6	12.5±7.0	0.03
Change in IOP (mmHg)			
0 hours	21.0±7.0	14.2±8.6	0.02
3 hours	−9.8±6.7	−7.2±12.0	0.6
6 hours	−1.9±8.0	−1.6±8.8	0.3
1 day	−5.7±9.6	−2.7±6.1	0.2
1 week	−4.2±5.1	−4.3±8.5	0.4
1 month	0.7±3.3	1.8±7.0	0.3

## Discussion

In this study, we showed that the presence of blocking air in the filtering bleb contributed to increased IOP early after DSAEK in eyes with previous TLE.

Several studies have reported an association between a history of filtering surgery and the graft detachment rate after DSAEK [7-12]. In a multivariate analysis of DSAEK cases, Nahum et al. [10] showed that previous TLE was an independent risk factor for postoperative graft detachment. Kang et al. [11] reported a high graft detachment rate (33.9%, 21 of 62 eyes) after DSAEK in eyes with previous TLE. Goshe et al. [12] reported that the graft detachment rate was significantly higher in eyes with previous filtering surgery (9%) than in the control group of eyes with Fuchs’ endothelial dystrophy (2%); moreover, graft dislocation was strongly associated with the presence of postoperative hypotony. These results indicated that the maintenance of sufficiently high IOP during the early postoperative period is essential for implanted graft adhesion to the stroma.

In our study, the graft detachment rate was significantly higher in eyes with a filtering bleb (14.3%) than in eyes without a filtering bleb (6.5%); the rate in eyes with a filtering bleb was an intermediate value between the graft detachment rate reported by Kang et al. [11] and that reported by Goshe et al. [12]. IOP and the amount of air in the AC were lower in eyes with a filtering bleb than in eyes without a filtering bleb during the early postoperative period. We presume that aqueous humor rapidly escaped into the filtering bleb in eyes with such a bleb; thus, the IOP required for graft attachment could not be maintained and poor graft adhesion was likely.

Several reports have been published regarding surgical procedures intended to maintain IOP during the early postoperative period in DSAEK cases with previous penetrating surgery. Banitt et al. [13] reported the injection of air from the AC until the subconjunctival space was filled, while Oyakawa et al. [14] injected ophthalmic viscoelastic devices into filtering blebs to obtain a sufficiently high IOP after DSAEK. These methods achieved good graft adhesion. Therefore, these studies indicated that the maintenance of high IOP within the first hour postoperatively is an important factor in graft adhesion success in DSAEK cases with previous penetrating surgery. Generally, air tamponade for 30-60 minutes postoperatively is particularly important for graft adhesion after DSAEK [15].

We focused on the presence of air in the filtering bleb at the end of the DSAEK procedure and compared early postoperative outcomes with and without air in the filtering bleb. To our knowledge, this is the first report concerning the behavior of air in the filtering bleb. Our study showed a significant difference in IOP enhancement between the preoperative measurement and the end of surgery when eyes were divided into two groups based on the presence or absence of air in the filtering bleb. Although it may be limited to a short duration after surgery, the presence of air in the filtering bleb (i.e., blocking air) may prevent aqueous humor leakage into the filtering bleb, resulting in the temporary maintenance of high IOP. However, we were unable to demonstrate a significant effect of blocking air in the filtering bleb on graft adhesion. Because the small population size of the group of eyes with air in the filtering bleb (group A1) may have affected the result, we are considering future analyses in a larger population.

There were several limitations in our study. First, the postoperative outcomes of DSAEK (e.g., visual acuity, endothelial cell counts, and complications) were not comprehensively evaluated; this was because we focused only on the presence of air in the filtering bleb, which affects graft detachment. Second, the flow of air injected into the AC at the end of surgery was not constant; however, a constant flow cannot be achieved with the current methods.

## Conclusions

The presence of a filtering bleb was associated with increased graft detachment compared with eyes without a filtering bleb. The presence of blocking air in the filtering bleb helps ensure increased IOP during the early postoperative period but has no significant effect on graft detachment rates.
